# A Profile Study of Japanese Encephalitis in an Industrial Hospital in Eastern India

**DOI:** 10.7759/cureus.38455

**Published:** 2023-05-02

**Authors:** Sangita D Kamath, Bijaya Jha, Tauheed Ahmed, Nilanjan Sarkar

**Affiliations:** 1 Internal Medicine, Tata Main Hospital, Jamshedpur, IND; 2 General Medicine, Tata Main Hospital, Jamshedpur, IND; 3 Radiology, Tata Main Hospital, Jamshedpur, IND

**Keywords:** neurological deficits, fever, vector, pigs, encephalitis

## Abstract

Introduction

Japanese encephalitis (JE), caused by a *Flavivirus*, is one of the common causes of mosquito-borne encephalitis the world over including India. The disease is endemic in many states of India, including Jharkhand. Mortality ranges from 30 to 40% in different studies and a large number of patients survive with permanent neuropsychiatric sequelae.

Aim

The study aimed to evaluate the clinical spectrum, laboratory (including radiological) features and outcomes of cases of JE admitted in our hospital.

Methods and materials

This is a retrospective observational study consisting of confirmed cases of JE admitted to the medical wards and critical care unit of Tata Main Hospital (TMH) from January to December 2022. The case records of patients were retrieved from Hospital Management System (HMS) and analysed for demographic characteristics, clinical presentations and treatment details along with outcome measures, which included length of stay (LOS), complications, and mortality.

Observation

Of the 14 confirmed cases, six (43.9%) were males and eight (57.1%) were females. The average age of male and female patients was 41.8 ± 23.1 and 35.1 ± 20.5 years respectively. A total of 35.7% of the patients were in the age group of 21 to 30 years. The clinical manifestations in the decreasing frequency were altered sensorium in 11 (78.6%) patients, headache in six (42.8%) patients, generalised convulsions in four (28.6%) cases, vomiting in three (21.4%) cases and weakness in all limbs and of the right half of the body in one (7.1%) case each. Objective neurological findings noted were neck stiffness (3, 21.4%), cog-wheel rigidity (3, 21.4%), tremors (2, 14.3%), delirium (2,14.3%), quadriparesis, facio-brachial dystonia and hemiparesis (1, 7.14%) patient each. Neutrophilic leucocytosis was observed in five (35.7%) patients and mild thrombocytopenia in two (14.3%) patients. The average C-reactive protein (CRP) level was 7.3 ± 6.6 mg/dL. Three (21.4%) patients had mild transaminitis. Cerebrospinal fluid analysis was abnormal in all patients with varying degrees of elevated protein and cell count, while adenosine deaminase (ADA) levels and sugar were normal in all patients. Magnetic resonance imaging (MRI) brain revealed bilateral thalamic T2 FLAIR (fluid-attenuated inversion recovery) hyperintensities in 11 patients (78.6%). The average length of hospital stay was 9.6 ± 4.7 days. Ten patients (71.4%) needed treatment in the critical care unit. Complications seen were acute respiratory distress syndrome (2, 14.3%), sepsis with multiorgan failure (2, 14.3%) and ventilator-associated pneumonia (1, 7.1%). The case fatality rate was one (7.1%). Six patients (42.9%) had residual neuropsychiatric sequelae.

Conclusion

JE continues to be a major health-related problem. Most cases are concentrated during the post-monsoon period, coinciding with a higher vector density. Patients from rural backgrounds were seen to be more susceptible. JE may present with varying severities of acute encephalitic syndrome. As there is no specific treatment, timely diagnosis is important to reduce the morbidity and mortality associated with this disease. Clinicians must be aware of the wide spectrum of presentation of this disease. A high degree of suspicion along with thorough clinical examination and appropriate investigations are needed to diagnose this condition early and prevent complications.

## Introduction

Japanese encephalitis (JE), a mosquito-borne flavivirus encephalitis, is one of the leading forms of viral encephalitis worldwide. It is a major public health problem, due to its potential for causing epidemics, high mortality, leaving behind neurological sequelae and affecting young productive populations. Since its isolation from Japan in 1943, it has spread worldwide and is now most prevalent in South Asia, South-East Asia and the Western Pacific region. In South-East Asia, there are estimated 50,000 cases and 15,000 deaths annually, predominantly affecting children less than 10 years [1].

It is caused by the Japanese encephalitis virus (JEV), an enveloped, single-stranded RNA virus belonging to the family *Flaviviridae *and genus *Flavivirus*. The virus contains three structural proteins - core protein (C), membrane protein (M) and glycosylated envelope protein (E), along with seven non-structural (NS) proteins - NS1, NS2A, NS2B, NS3, NS4A, NS4B and NS [2]. It is a neurotropic virus. After crossing the blood-brain barrier, it replicates in the neurons, sets in a perivascular inflammatory reaction and causes neurolysis and microglial proliferation. These pathological changes affect the thalamus, cerebrum, brain stem, anterior horn cells of the spinal cord and cerebellum, which ultimately produce cerebral oedema, brain-stem herniation and death.

In India, the virus was first isolated in humans from the Vellore district of Tamil Nadu, in 1955. In 1973, large outbreaks were described in the districts of Burdwan and Bankura in West Bengal, where 300 and 700 deaths respectively were reported. Since then, outbreaks have been reported from several states of India like Uttar Pradesh, Karnataka, Kerala, Odisha, Andhra Pradesh, Assam, Bihar, Jharkhand and Tamil Nadu. The trend of JE suggests that the problem is escalating in India and larger epidemics may occur in future.

JEV is transmitted to humans through the bite of infected mosquitoes of the Culex species (mainly Culex tritaeniorhynchus, Culex vishnui group in India). The virus exists in an enzootic transmission cycle between mosquitoes, pigs and/or water birds. In Asia, pigs are the amplifying host, providing a link to humans through their proximity to housing. Rural areas closely linked to paddy fields and piggeries are considered to be high-risk areas for disease transmission. In India, transmission occurs all year round with increased cases observed during the rainy season and pre-harvest period in rice-cultivating regions.

Most of the cases are mild or asymptomatic. The disease manifests mainly in children and young adults with high-grade fever, myalgias, headache and vomiting followed by various neurological symptoms like altered sensorium, spastic paralysis, seizures, coma and death in severe cases. The incubation period varies from six to 14 days. The initial presentation in children usually begins with gastrointestinal symptoms like abdominal pain, nausea and vomiting. The case-fatality ratio described in the literature is as high as 42% with high mortality in children less than 10 years and adults more than 65 years [3]. Twenty to fifty per cent of the survivors develop permanent neurological sequelae like intellectual, behavioural and neurological deficits like Parkinsonism, speech disturbances, paralysis and ataxia [3,4]. Diagnosis is established by testing for JEV-specific IgM antibodies in a single sample of cerebrospinal fluid (CSF) using an IgM-capture ELISA. There is no specific antiviral agent for JE. Treatment is largely supportive.

Every year, sporadic cases have been reported from Jharkhand, indicating the endemicity of the disease in the state. Despite this, data especially regarding the clinical profile of adult cases of JE from this part of the state is limited. In light of this, we undertook the present study at Tata Main Hospital (TMH) with the aim of studying the clinical spectrum, laboratory (including radiological) features and outcomes of cases of JE.

## Materials and methods

This was a retrospective observational study. Patients who fulfilled the following inclusion criteria from January to December 2022 were included. The study population constituted patients admitted to the medical wards and critical care unit (CCU) of Tata Main Hospital (TMH), Jamshedpur, which is a 983-bedded industrial, tertiary care hospital in Jharkhand. The study was cleared by the Institutional Ethics Committee (IEC).

Inclusion criteria

1. Patients presenting with acute encephalitis syndrome (AES), characterized by acute onset of high fever, altered mental status and/or new onset of seizures as per WHO guidelines.

2. Age 15 years and above

3. JE IgM antibody positive in CSF

Exclusion criteria

1. Age < 15 years

2. CSF/serum negative for JE IgM antibody

Methodology

Detailed records of all patients including demographic characteristics like age, sex, presenting clinical features, past history, and personal and family history were noted from the medical records retrieved from the hospital management system. Findings of a complete physical examination, including a detailed neurological examination and progress, were noted. Routine blood investigations like complete blood picture including platelet count, liver function and renal function tests, CSF findings for protein, sugar, cells, bacteria and fungi, along with findings of radiological investigations were noted. The outcomes observed included the length of stay (LOS), response to therapy, neurological deficit, duration of critical care unit (CCU) stay, duration of ventilator days, complications related to various organs and mortality.

Sample collection and antibody testing

CSF samples of suspected cases were collected and sent from our hospital to the virology unit of the Microbiology Department of Mahatma Gandhi Memorial (MGM) Medical College and Hospital, Jamshedpur maintaining a cold chain. All the samples were tested for IgM antibodies with an IgM Antibody Capture (MAC) ELISA Kit supplied by the National Institute of Virology (NIV), Pune JE as a part of the National Vector Borne Disease Control Program (NVBDCP). If the optical density (OD) value of the tested sample was more than that of the negative control by a factor of five, the sample was considered positive for IgM. The test had a diagnostic sensitivity of 95% and a specificity of 98%.

Statistical analysis

Categorical variables were expressed as numbers and percentages, while continuous variables were described in terms of means, medians, ranges and standard deviations (SDs). As the total number of cases was small, the tests for statistical significance were not used and the P value was not calculated.

## Results

**Figure 1:**
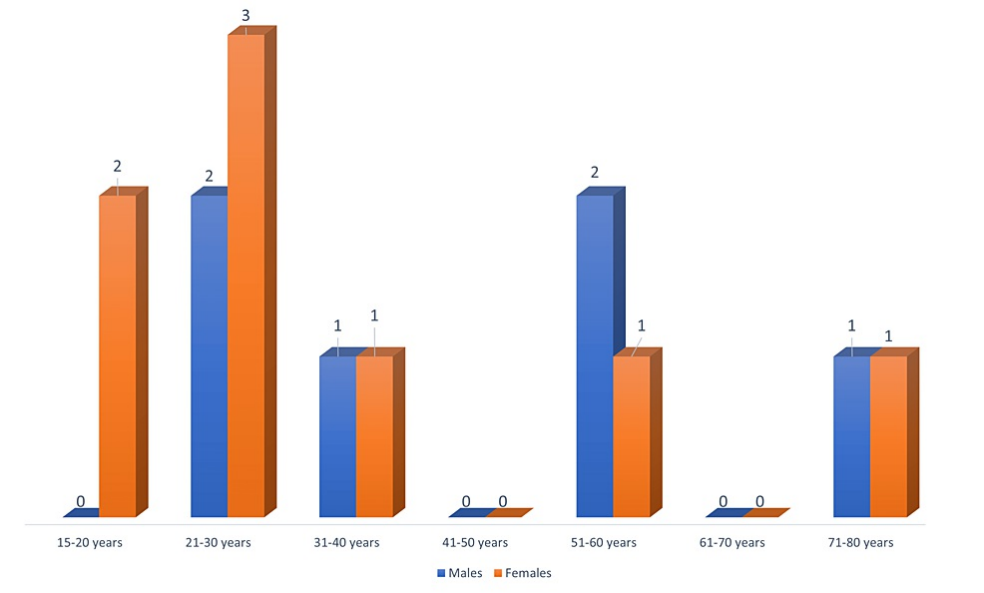
Age and sex distribution of the cases of JE (n=14) JE: Japanese encephalitis

Of the total 42 cases of AES, 14 cases were those of confirmed JE, giving rise to case positivity of 40.5%. Of these, three were below 15 years and were excluded. Six (43.9%) were males and eight (57.1%) were females with male to female ratio being 0.75:1 as shown in* *Figure 1. Their age ranged from 15 to 75 years, with the mean age (±SD) being 38 (±21.2) years as shown in Figure 1. The average age of males was 41.8 ± 23.1 years, while that of females was 35.1 ± 20.5 years. A total of 35.7% of the patients were in the age group of 21 to 30 years.

**Figure 2:**
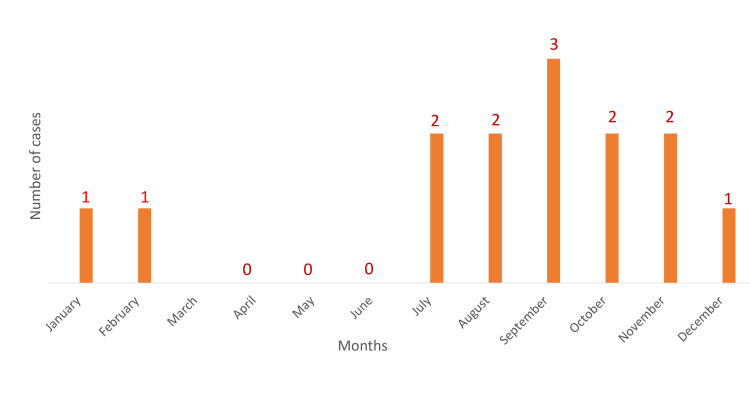
Distribution of cases month-wise (N=14)

Nine out of 14 patients (64.3%) were admitted in the months of July to October (Figure 2) indicating a propensity for monsoon months when the vector density is higher.

**Figure 3:**
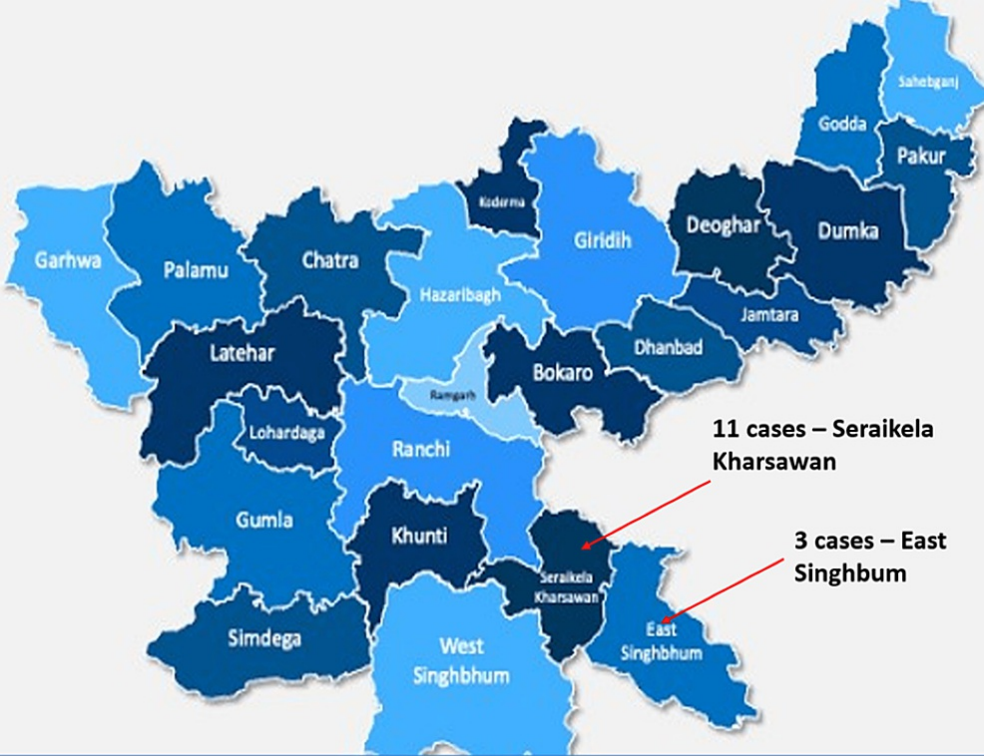
Geographical distribution of admitted cases (N=14)

Most of the cases (11, 78.6%) were from the Seraikela-Kharaswan district of Jharkhand (Figure 3) and were illiterate or had only primary school education with a rural background.

Clinical features

**Figure 4:**
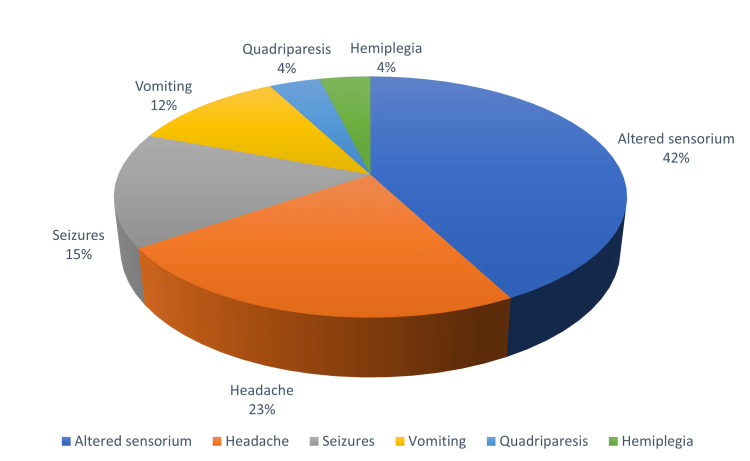
Presenting symptoms on admission (N=14)

Fever was the commonest presenting symptom seen in all the patients. It was associated with chills and rigours. The average duration of fever was 5.1 ± 2.8 days. Other symptoms in the decreasing order were altered sensorium in 11 (78.6%) patients, headache in six (42.8%) patients, generalised convulsions in four (28.6%) cases of which one (7.14%) patient had focal convulsions, vomiting in three (21.4%) cases and weakness of all limbs in one (7.14%) and of the right half of the body in one (7.1%) case (Figure 4). Sensorium which was assessed by Glasgow Coma Scale (GCS) varied from 4/15 to 9/15. Objective neurological findings were seen in all patients. Two (14.3%) patients were in delirium, three (21.4%) had extra-pyramidal features like cog-wheel rigidity, and tremors were seen in two (14.3%) patients. Quadriparesis, facio-brachial dystonia and hemiparesis were seen in one (7.14%) patient each. Neck stiffness was noted in three (21.4%) patients. Two (14.2%) patients were in septic shock at the time of admission.

Laboratory investigations

The average haemoglobin level was 10.2 ± 1.4 g/dL, while the average platelet count was 187,214 ± 118,285/mm^3^. Mild thrombocytopenia was seen in two (14.3%) patients while thrombocytosis (platelet count of 5.43 lakh/cu mm) was observed in one (7.1%) patient. The lowest leucocyte count seen was 4,000/mm^3^, while the highest count was 17,400/mm^3^. The average leucocyte count was 9,992 ± 4,283.01/mm^3^. Neutrophilic leucocytosis was observed in five (35.7%) patients while the total WBC was within normal limits in nine (64.3%) patients. The average C-reactive protein (CRP) level was 7.3 ± 6.6 mg/dL, while the highest level observed was 23.4 mg/dL. Hypernatremia was seen in two (14.3%) patients. Three (21.4%) patients had a mild elevation of liver enzymes, of which one (7.1%) patient had an elevation more than five times the upper limit of normal (ULN). Serum creatinine was normal in all patients on admission except in those who had underlying chronic kidney disease (CKD).

CSF analysis

CSF was colourless in all patients. The total leucocyte count was increased in all 14 cases (100%) with a mean of 67.8 (± 106.5) cells per mm^3^. The minimum WBCs noted were 8 cells/cu mm while the maximum noted were 380 cells/cu mm. One hundred per cent lymphocytes were seen in 11 patients (78.6%) while neutrophilic predominance was noted in three (21.4%) cases. CSF protein was increased in all 14 cases (100%) with a mean of 100.76 (± 27.28) mg/dL. Glucose and adenosine deaminase (ADA) levels were normal in all cases. Mean glucose and ADA levels were 73 ±14 mg/dL and 3.87 ±1.13 IU/L respectively. IgM ELISA for JE virus antibody was positive in all patients.

Neuroimaging

**Figure 5:**
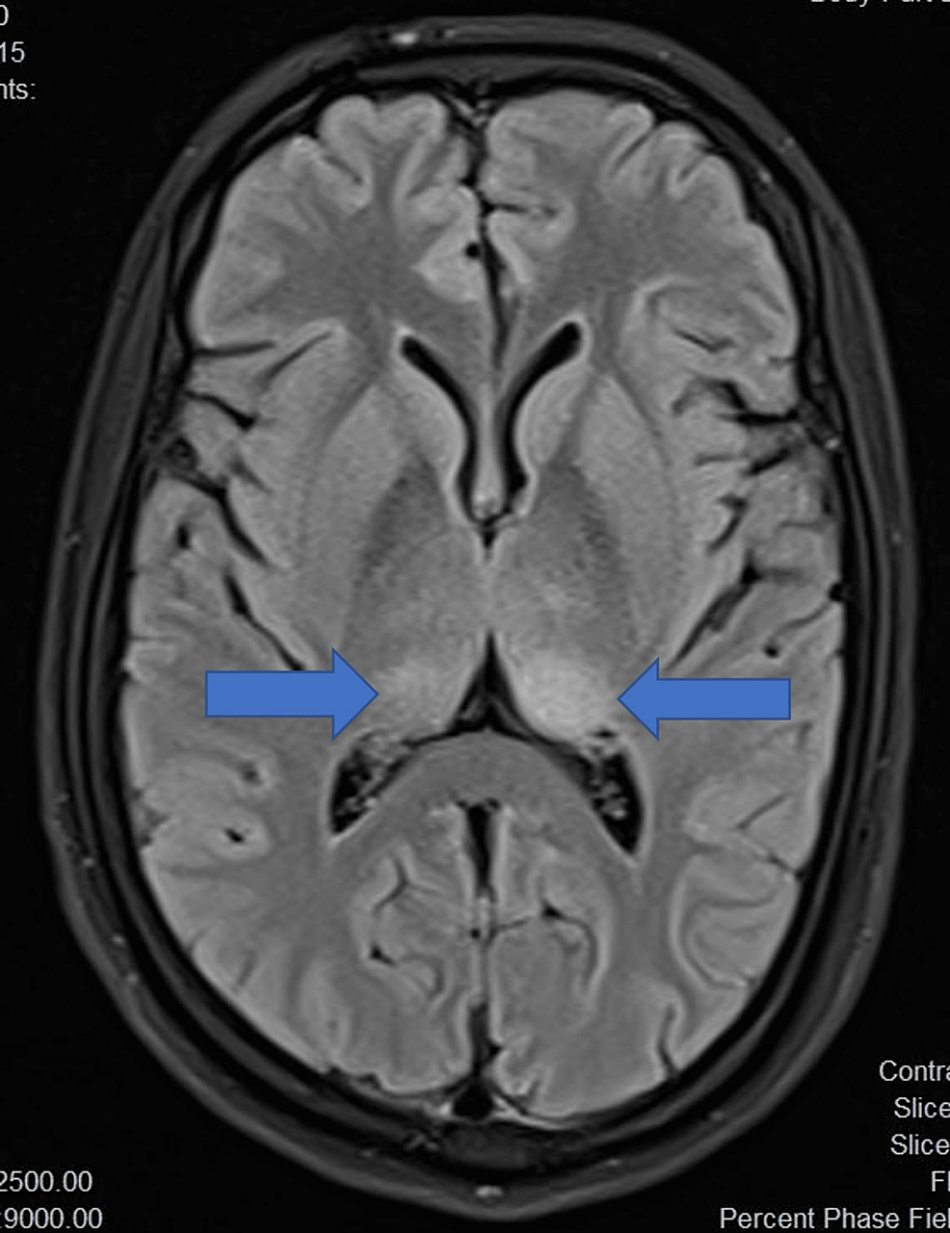
T2 FLAIR axial MRI scan showing bilateral nearly symmetrical hyperintensities in the thalamus FLAIR: fluid-attenuated inversion recovery; MRI: magnetic resonance imaging

**Figure 6:**
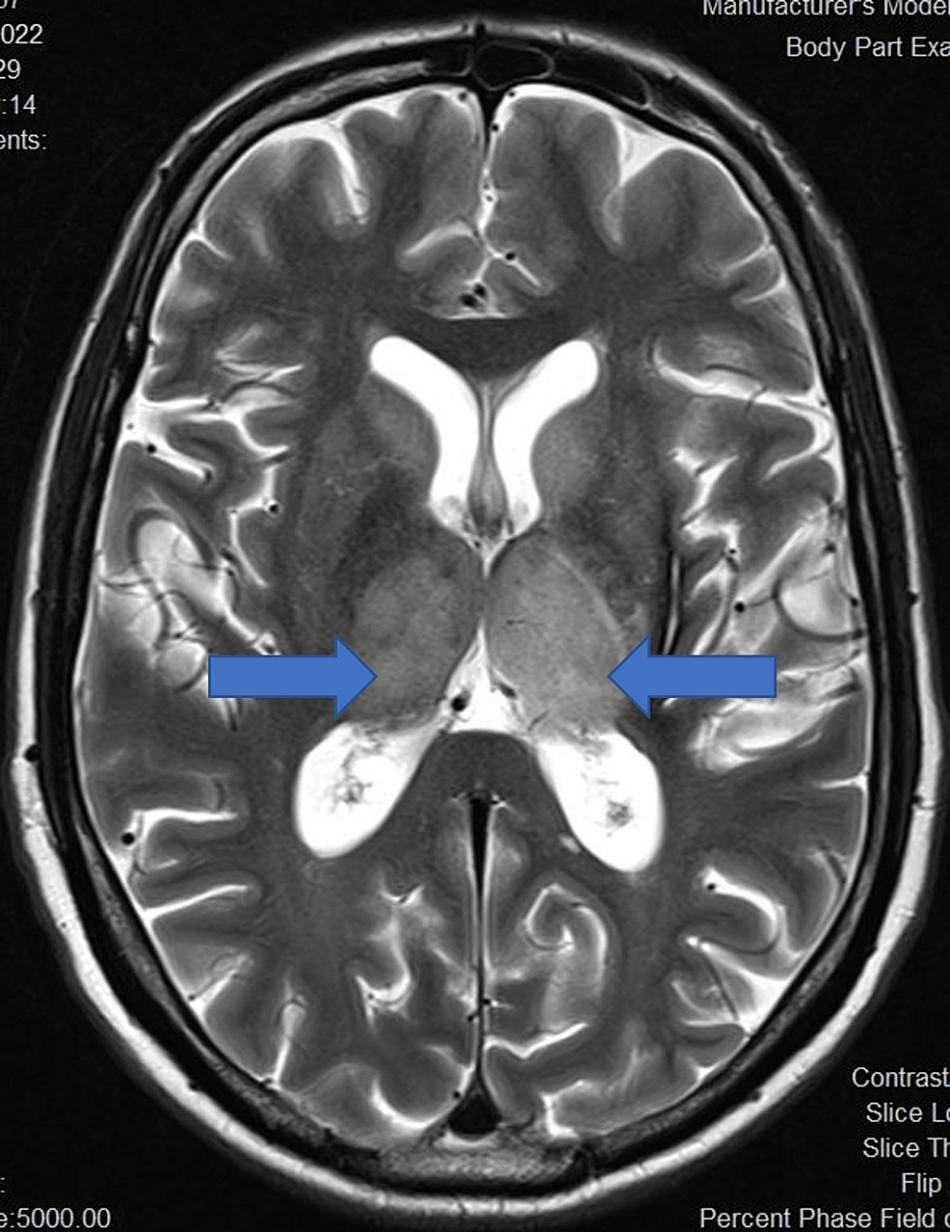
T2 axial MRI image showing bilateral enlarged thalami with hyperintensity MRI: magnetic resonance imaging

**Figure 7:**
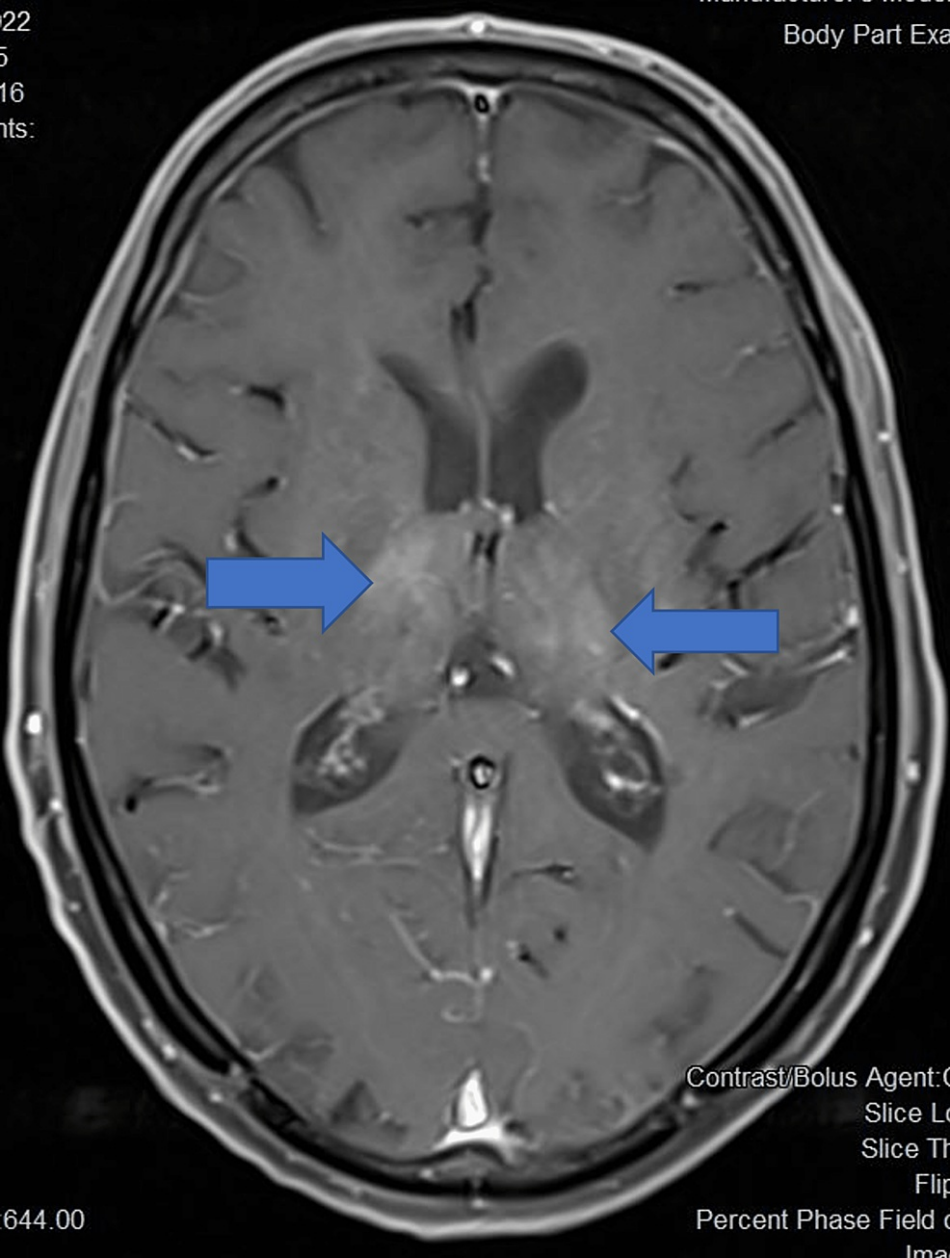
Post-contrast T1 axial image shows patchy enhancement in bilateral thalami

**Figure 8:**
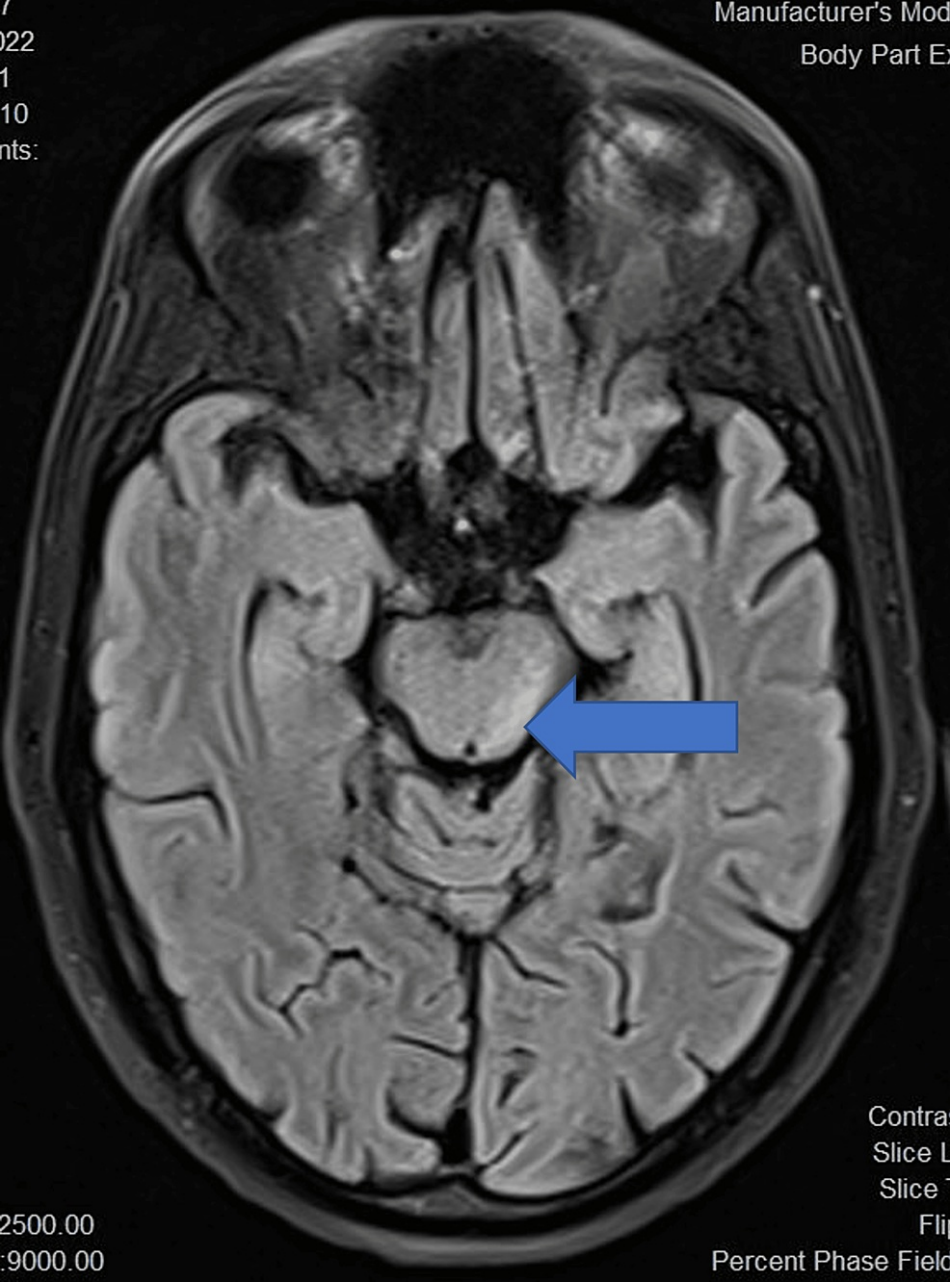
T2 FLAIR axial image shows patchy hyperintensity in midbrain FLAIR: fluid-attenuated inversion recovery

Initial neuroimaging was done using a non-contrast computed tomography (NCCT) brain for all patients. NCCT brain was normal in eight out of 14 (54.1%) patients while cerebral oedema was noted in five patients (35.7%). Magnetic resonance imaging (MRI) brain revealed bilateral thalamic T2 FLAIR (fluid-attenuated inversion recovery) hyperintensities in 11 patients (78.6%) (Figures 5-7), of which four (28.6%) also had involvement of multiple areas including midbrain (Figure 8) and basal ganglia. Patchy leptomeningeal enhancement was noted in two patients (14.3%). Multiple, focal, micro bleeds were seen in two (14.3%) patients.

Outcomes

All patients were treated with symptomatic supportive treatment. The average length of hospital stay was 9.6 ± 4.7 days. Of the 14 patients, 10 (71.4%) needed treatment in the critical care unit, while four (28.6%) were managed in the medical wards. Five patients (35.7%) developed complications like ARDS - acute respiratory distress syndrome (2, 14.3%), sepsis with MODS - multiorgan dysfunction syndrome (2, 14.3%) and VAP - ventilator-associated pneumonia (1, 7.1%). One patient expired giving rise to the case fatality rate of 7.1% and two patients were discharged against medical advice (DAMA). The rest of the patients were discharged in stable condition. However, six patients (42.9%) had residual neurological sequelae which included tremors in two (14.3%) patients, varying degrees of paralysis in three (21.4%) patients and cognitive impairment in two (14.3%) patients.

## Discussion

In India, epidemics of JE have been reported from time to time in many parts of the country, and it continues to be a major public health problem. Approximately, 60 crores people in India live in JE endemic areas and 1,500 to 4,000 cases are reported every year, with many cases going unreported [1]. It is an important cause of AES throughout the world and particularly in Asia. Spread across the geographic areas has been facilitated by the combined effects of international travel, migration of birds and animals, deforestation and development of irrigation projects. Five genotypes (G-I to G-V) of JEV have been identified by nucleotide sequencing studies [2].

A study of the seasonal distribution of cases revealed higher incidence from July to December, with 78.6% of cases being admitted in the months of July to November, while no cases were detected from March to June. Similar findings were noted by Bandyopadhyay B et al. [4], Anuradha SK et al. [5] and Sarkar A et al. [6]. This may be explained by the rural background of our patients and the increased prevalence of the Culex mosquitoes which breed abundantly on the paddy fields covered with stagnant water during the rainy season.

There was a slight female preponderance of the cases in our study. Similar results were found in a study by Bandyopadhyay B et al. from West Bengal [3]. A study conducted by Borah J et al. [7] from Assam revealed a male preponderance of the cases. However, in a four-year study from Jharkhand by Sinha N et al. [8], more cases were reported in females in 2018 and 2019, while a male preponderance was seen in 2010 and 2021. Also in 2021, out of the 54 confirmed cases, most were children and adolescents. Maximum cases occurred in the 6-10 years of age group (29, 53.7%) followed by 11-15 years (15, 27.8%) and 10 cases (18.5%) in zero to five years of age. Bandyopadhyay B et al. in their study observed that 48.21% and 61.11% of JE-positive cases were below the age of 20 years in 2011 and 2012, respectively [4]. Our study excluded paediatric patients. A total of 28.6% of our patients were in the age group of 21 to 30 years. We also came across five (35.7%) cases beyond the fifth decade of life. In a study from northern Thailand by Gould DJ et al. [9], the incidence was around 40 cases per 1,00,000 in the age group of five to 25 years decreasing to almost zero for those above 35 years.

In our study, the clinical picture was characterised by fever in all patients followed by altered sensorium in 11 (78.6%) patients. Convulsions were seen in four (28.6%) cases of which one (7.1%) had focal facio-brachial seizures, while three (21.4%) had Parkinsonian features like cog-wheel rigidity, and tremors were seen in two (14.3%) patients. In a study by Misra UK et al. [10] from Lucknow, of the 14 cases, seizures were reported in seven (50%) patients, Parkinsonism features in eight (57.1%) patients, movement disorders like dystonia in five (35.7%) and chorea in one (7.1%) patient. Eleven (78.6%) patients had quadriplegia and one (7.1%) patient had hemiplegia. In our series too, one patient (7.1%) each had quadriplegia and hemiplegia. Verma A et al. [3] reported tremors in six (42.8%) cases, dystonia in two (14.2%), emotional lability in two (14.2%), and right-sided weakness in one (7.1%) case in a study involving paediatric patients from Jharkhand. In yet another study by Borah J et al. [7] comparing clinical features of JE infection in the adult and paediatric age groups, fever was seen in all adult cases as in our study. Headache was seen in 87.2% of cases, neck rigidity in 55.2%, convulsions in 37.8% and abnormal behaviour in 22.7% of adult cases. Neck rigidity, seizure and abnormal behaviour were significantly higher in children (P < 0.05) than in adults.

Three (21.4%) patients in our study had an elevation of liver enzymes, while 93 (54.15%) patients had an elevation of alanine transaminase (ALT) (mean level ± SD, 76.4 ± 42.3U/L), 48 (27.9%) patients had an elevation of aspartate transaminase (AST) (mean level ± SD, 115.3 ± 44.6U/L) and four (2.3%) patients had serum bilirubin > 1mg/dL (mean ± SD, 1.3 ± 0.33) in the study by Borah J et al. Thus, the elevation of both liver enzymes was seen in their study as against the study by Solomon T et al. [11], who observed elevated AST (55 ± 44.2) but normal ALT levels in adults. Blood urea and serum creatinine were normal in all our patients except one who had underlying CKD. This is similar to that reported in the literature, except in a study done from Uttar Pradesh, which reported elevated blood urea levels in three out of 77 paediatric patients [12].

In our study, neutrophilic leucocytosis was observed in five (35.7%) patients, with the average leucocyte count being 9,992 ± 4,283.01/mm^3^, while mild to moderate polymorphonuclear leucocytosis (12,000 to 40,000/cu mm) was observed in 89.83% of the patients in a study by Sarkari NB et al. [13] from Gorakhpur. However, leucocytosis >11,000 cells/mm^3^ was observed in 56 (32.6%) patients with a mean value of 13,241 ± 5319/mm^3^ and thrombocytopenia (platelets less than 10^5^/mm^3^) was noted in 35 (20.3%) adult patients in the study by Borah J et al. [7]. Mild thrombocytopenia was seen in two (14.3%) patients in our study. Thrombocytosis was seen in one (7.1%) of our patients and has not been reported in other studies.

Abnormalities in CSF were seen in all our patients (100%) with an increase in protein and cell count with lymphocytic predominance. In a study by Borah J et al. [7] involving 172 adult patients of JE from Assam, the mean level of CSF protein was 72.7 ± 46.9 mg/dL, while that of white blood cells (WBC) was 69.4/cu mm ± 23.6. In a Vietnamese study by Solomon T et al. [11], 112 patients (77.8%) had CSF pleocytosis (range from 0 to 550 WBC/cu mm, median 53). Sixty-four (44.5%) had lymphocytic while 27 (18.8%) had neutrophilic predominance. However, the CSF picture did not influence the outcome. In a study of 14 patients by Misra UK et al. [10], CSF was found to be abnormal in nine (64.3%) patients, while five (35.7%) patients had normal findings. The mean CSF protein was 82.6 mg/dL (range 16-397), average sugar was 63.3 mg/dL (range 10-150) while the mean cell count was 73 (range 0-930)/mm^3^ with lymphocytic predominance in 72% patients.

Neuroimaging with NCCT brain revealed abnormalities in five (35.7%) patients, while MRI brain was abnormal in 13 patients (92.8%). Our findings were similar to those in a study by Kalita et al. [14] in patients with JE. In their study, abnormalities were seen in 38.7% of adult patients with JE on CT and 90.6% of adult patients on MRI. The most common areas involved in MRI included the thalamus (87.5%) and basal ganglia (40.6%). Less common involvement of the cortex (21.9%), midbrain (28.1%) and pons (9.3%) were also seen. In another study by Misra UK et al. [10] among 14 adults with JE, MRI showed abnormalities in 11 (78.6%) patients. Thalamus and basal ganglia were most commonly involved in 10 (71.4%) and seven (50%) patients respectively. Involvement of the brainstem was seen in seven (50%) patients, and the cortex was involved in two patients (14.3%). In a systemic review by Pichl T et al. [15], thalamic lesions were the most reported MRI abnormality in JE (74% in 23 studies). However, these findings were not pathognomonic as they were seen in a significant proportion of dengue cases also. In another systematic review by Aryal R et al. [16], the most common site of lesions on MRI was the thalamus, followed by the basal ganglia, brainstem, cortex and substantia nigra. One (7.1%) patient in our study had a rare finding of leptomeningeal enhancement around the brain stem.

Six (42.9%) patients in our study had residual neurological sequelae which included extrapyramidal symptoms like tremors, varying degrees of paralysis and cognitive impairment. In a study by Basumatary LJ et al. [17], among 104 adults with JE, 54.8% suffered from neurological sequelae at the time of discharge, with dystonia and Parkinsonian features being the most common. Also, in a study from Jharkhand by Varma A et al., 10 (71.4%) patients had sequelae the most common being speech problems in 50% of cases, followed by tremors (42.8%), dystonia (14.2%), emotional lability (14.2%) and right-sided weakness (7.1%) [3]. In the largest study from (Gorakhpur) UP by Sarkari NB et al., covering four epidemics of JE involving 645 confirmed cases, neurological sequelae were found in a high percentage of cases. Speech disturbances were observed in 79.4% of cases, extra-pyramidal features in 88.6% of cases, residual paralysis in 74.4% of cases, dystonic postures in 7.13% of cases and cranial nerve deficits in 32.03% of cases. Only 3% of their patients did not have any neurological deficits [13].

The mean duration of hospital stay in our study was 9.6 ± 4.7 days. One patient expired giving rise to the case fatality rate of 7.1%. The patient was a female of 32 years who presented with a GCS of 4/15 and had three episodes of generalised seizures on admission. Our case fatality was similar to that reported from Jharkhand by Verma A et al. [3] but with a higher average length of stay of 16.14 days. Dwibedi B et al. [18] from Odisha reported case fatality care of 8.8% with a mean recovery period of seven days. The non-neurological complications observed in various studies [13,7] were pulmonary oedema (33.3%), upper gastrointestinal bleed (15.8%), pericarditis (8.3%), septic shock (5.1%), transaminitis (54.2%) and renal dysfunction (in form of raised blood urea) in 11.6% cases. In our study, we observed septic shock, ARDS, VAP and mild transaminitis. None of our patients had received vaccination against JE.

This study highlights the need for increased awareness of JE amongst clinicians in Jharkhand. We suggest that the diagnosis of JE should be based on a high index of suspicion, thorough clinical evaluation and a constellation of laboratory parameters including imaging, as mentioned above. As the disease is associated with high morbidity and mortality, there is a need to ramp up surveillance and implement a robust immunization programme in Jharkhand.

## Conclusions

JE is a major public health problem. It is prevalent in this part of the country and should be considered in the differential diagnosis of a patient presenting with acute febrile illnesses and altered sensorium (AES). Its wide spectrum of clinical presentation along with the complexity of diagnosis makes it difficult to diagnose early. Lack of specific treatment along with high case fatality and abundance of neurological sequelae contributes to the disease burden. In the absence of specific treatment, early suspicion and supportive treatment may limit the morbidity and mortality associated with the disease. Implementation of a vaccination program along with rigorous surveillance systems and vector control are the only ways to limit the spread of the disease and prevent outbreaks.
